# Determination of reference genes as a quantitative standard for gene expression analysis in mouse mesangial cells stimulated with TGF-β

**DOI:** 10.1038/s41598-022-19548-z

**Published:** 2022-09-17

**Authors:** Bruno Aristides dos Santos Bronel, Ana Carolina Anauate, Edgar Maquigussa, Mirian Aparecida Boim, Antônio da Silva Novaes

**Affiliations:** Renal Division, Department of Medicine, Universidade Federal de São Paulo, Rua Pedro de Toledo, 781, São Paulo, SP 04039-032 Brazil

**Keywords:** Molecular medicine, Nephrology

## Abstract

Reverse transcription-quantitative polymerase chain reaction (RT-PCR) is the gold standard technique for gene expression analysis, but the choice of quantitative reference genes (housekeeping genes, HKG) remains challenging. Identify the best HKG is essential for estimating the expression level of target genes. Therefore, the aim of this study was to determine the best HKG for an in vitro model with mouse mesangial cells (MMCs) stimulated with 5 ng/mL of TGF-β. Five candidates HKG were selected: *Actb, Hprt, Gapdh, 18S* and *Ppia*. After quantitative expression, the best combination of these genes was analyzed in silico using six software programs. To validate the results, the best genes were used to normalize the expression levels of *fibronectin*, *vimentin* and *α-SMA*. In silico analysis revealed that *Ppia*, *Gapdh* and *18S* were the most stable genes between the groups. GenEX software and Spearman's correlation determined *Ppia* and *Gapdh* as the best HKG pair, and validation of the HKG by normalizing *fibronectin*, *vimentin* and *α-SMA* were consistent with results from the literature. Our results established the combination of *Ppia* and *Gapdh* as the best HKG pair for gene expression analysis by RT-PCR in this in vitro model using MMCs treated with TGF-β.

## Introduction

Transforming growth factor-β (TGF-β) is a major renal profibrogenic cytokine and plays a critical role in mesangial dysfunction in many pathophysiological conditions characterized by excessive accumulation of extracellular matrix (ECM) proteins, mesangial cell (MCs) hypertrophy, and proliferation^1,2^. The interaction of TGF-β and its receptors forms a heterodimeric complex, which is translocated into the nucleus and regulates transcription of target genes, such as *fibronectin*, *vimentin* and *α-smooth muscle actin* (*α-SMA*)^1,3–5^.

The reverse transcription-quantitative polymerase chain reaction (RT-PCR) is the gold standard method to identify changes in mRNA expression levels^6–8^. Considering the many steps of RT-PCR, and that several factors can influence expression levels, the normalization of target genes is crucial for accurate gene expression quantification. Currently, the most accepted method of target gene expression normalization by RT-PCR technique is through quantification of very stable endogenous housekeeping genes (HKG)^9,10^.

HKG or reference genes, are genes required for maintenance of basal cellular functions^11,12^ and the ideal reference genes are expected to be expressed in all cell types and should show minimal variation in the expression, regardless of cell cycle state, developmental stage, external stimuli and physiological condition^10,12,13^. Examples of the most used reference genes include *Actin beta* and *Gapdh*^14^. Although the use of HKG is the most accurate method for normalizing mRNA expression levels, it is well known that the expression levels of even the most stable HKG can change depending on cell type and experimental conditions and design^8,15^.

Despite the growing number of studies investigating the reference genes for renal disease models^10,16,17^, to our knowledge there are no detailed reports selecting the most stable gene recommended for the frequently used in vitro model of TGF-β-induced fibrogenesis in MCs. This limitation may lead to non-reproducible data. Therefore, the present study aimed to evaluate the performance of five frequently used reference genes (*Actb, Hprt, Gapdh, 18S* and *Ppia)* in renal models^18–29^ and to identify the most stable ones and the optimal number of genes for normalization the expressions of target genes by RT-PCR in MCs treated with TGF-β model.

## Results

### Candidate housekeeping genes

**Figure 1:**
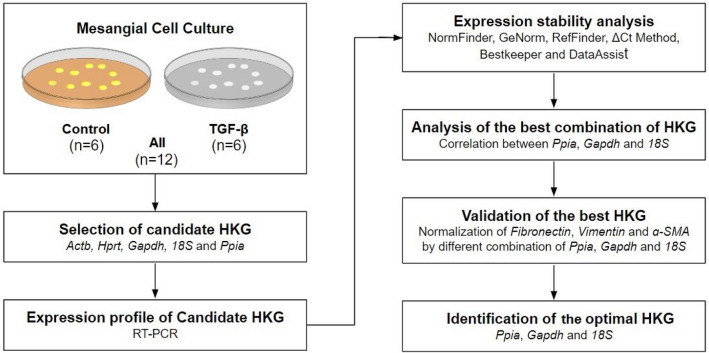
Workflow diagram illustrating the strategy for identification of housekeeping normalizer for RT-PCR. *Actb, Hprt, Gapdh, 18S* and *Ppia* housekeeping genes (HKG) selected from the literature for comparison.

**Figure 2:**
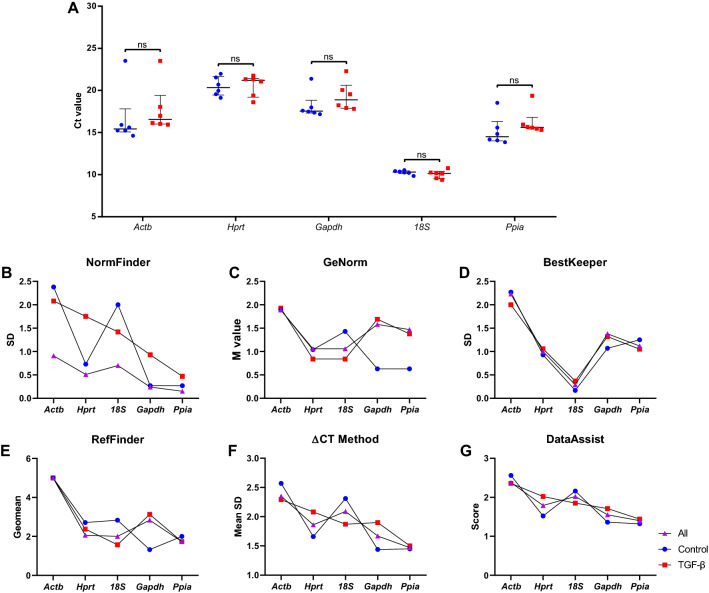
Ct values of five candidate housekeeping genes (**A**) and stability analysis of housekeeping genes by six different software (**B–G**). A lower cycle threshold (Ct) value indicates a higher gene expression (**A**). The median values are expressed as horizontal lines, and the error bars represent the interquartile range. The Ct values of *18S* were the lowest, indicating the highest expression levels. Best housekeeping gene for each group of samples yielded by software analysis (**B–G**). All, all samples. ns, non-significant.

First, we followed a stepwise strategy to identify the best HKG expression by RT-PCR analysis. The workflow diagram is shown in Fig. 1. The samples were classified into three groups: (1) control cells (n = 6); (2) cells treated with TGF-β (n = 6); and (3) All, which includes all cells (n = 12). The Ct values of the five candidate HKG ranged between 23.511–9.387 and are expressed as [median (interquartile range)]. Ct values are inversely proportional to gene expression, and the mean highest Ct value among the candidate genes was achieved by *Hprt* [20.876 (2.05)], indicating the least expressed gene. In contrast, the lowest Ct value was obtained for *18S* [10.232 (0.50)], indicating the highest expression among the candidate genes. *Gapdh* [17.948 (2.41)], followed by *Actb* [15.986 (2.44)] and *Ppia* [15.514 (1.54)], showed moderate expression. The median Ct values of triplicate reactions according to each gene is shown in Fig. 2A. Furthermore, no statistically significant differences were found between the control group and the TGF-β-treated group. Thus, these data suggest that the endogenous genes selected showed good stability and that their expression did not change when treated with TGF-β.

### Stability analysis of housekeeping genes

Stability analysis of the five candidate HKG were determined using six software packages (Supplementary Table 1). Genes with the smallest stability value (SV) had the most stable expression. Following NormFinder criteria (SD < 0.5), only *Ppia* and *Gapdh* had a SD value below 0.5 in the All and control groups, while in the TGF-β-treated group, only *Ppia* respects this criterion (Fig. 2B and Supplementary Table 1). The GeNorm software defines an M value < 1.5; therefore, the genes with lower variability were: *Hprt*, *18S* and *Ppia* for All samples; *Gapdh*, *Ppia*, *Hprt* and *18S* for the control group; and *18S, Hprt* and *Ppia* for the TGF-β-treated group (Fig. 2C and Supplementary Table 1). According to Bestkeeper criteria, when considering all three experimental groups, only *18S* fits the parameters, although the CV exceeds 3.0 in the TGF-β group (Fig. 2D and Supplementary Table 1).

In the RefFinder and ∆Ct method analysis, the most stable gene in the All group was *Ppia*, while in the control group it was *Gapdh*. However, in the TGF-β group, the most stable genes were *18S* and *Ppia* according to RefFinder and ∆Ct method analysis, respectively (Fig. 2E–F and Supplementary Table 1). The evaluation of the most stable gene on DataAssist software identified that *Ppia* as the best HKG in the All, control, and TGF-β-treated groups (Fig. 2G and Supplementary Table 1). In all software analysis, the less stable gene was *Actb* (Fig. 2B–G and Supplementary Table 1). Based on software analysis and a qualitative inspection of all ranks generated, the best HKG for the All group was *Ppia*, whereas it was *Gapdh* for the control group and *Ppia* or *18S* for the TGF-β-treated group (Fig. 2B–G and Supplementary Table 1).

### Analysis of the best combination of housekeeping genes

**Figure 3:**
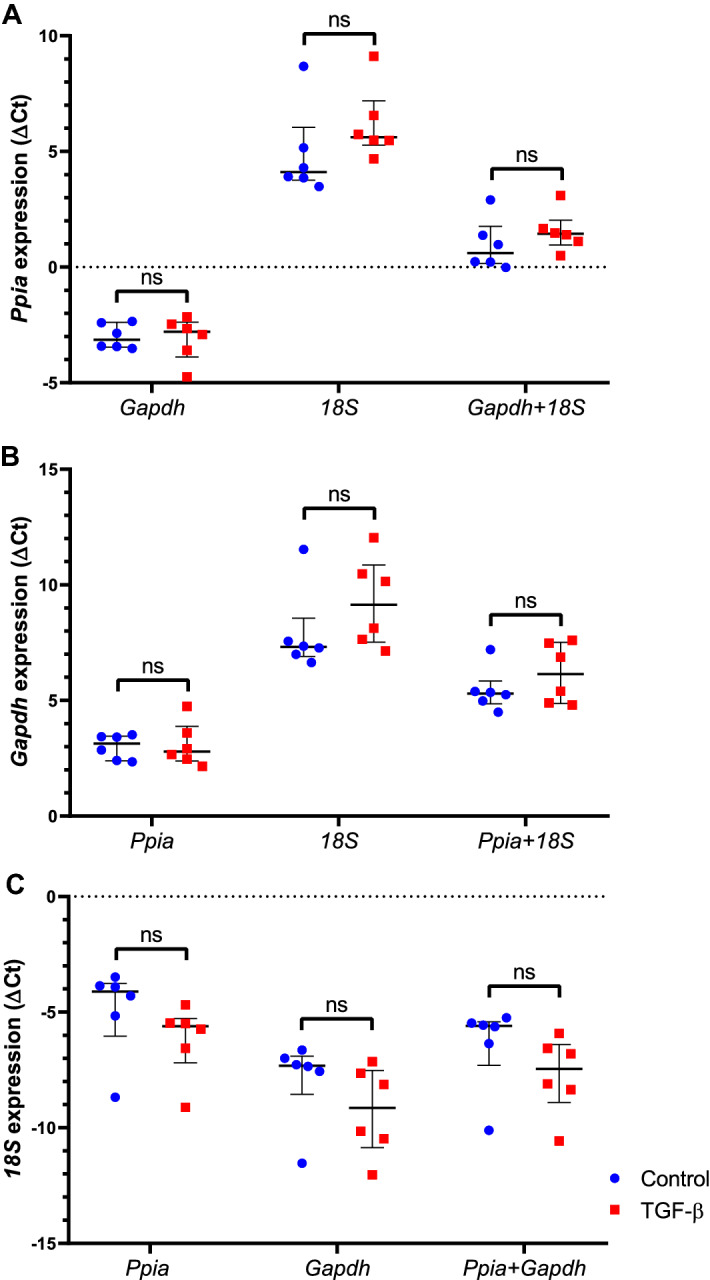
The ΔCt values of *Ppia* (**A**), *Gapdh* (**B**) and *18S* (**C**) candidate housekeeping genes were normalized by combinations of each other. A negative ΔCt value indicates that the target gene is more abundant than the HKG. The median values are expressed as horizontal lines, and the error bars represent the interquartile range. *Ppia*, target expression normalized by *Gapdh*, *18S* and *Gapdh* + *18S*; *Gapdh*, target expression normalized by *Ppia*, *18S* and *Ppia* + *18S*; *18S*, target expression normalized by *Ppia*, *Gapdh* and *Ppia* + *Gapdh*. ns, non-significant.

The GeNorm software recommends at least two genes for gene expression normalization, and the best combination of HKG for each group/software package is shown in Table 1. In the All group, the best pair of HKG is *Ppia* + *Gapdh* or *Ppia* + *18S*; in the control group is *Gapdh* + *Ppia* and in the TGF-β group is *Ppia* + *18S* (Table 1). To analyze the effects of the best candidate HKG, the expression levels of the top three (*Ppia*, *Gapdh*, and *18S*) were normalized by each other (Fig. 3). All comparisons showed no statistically differential expression, meaning that *Ppia*, *Gapdh*, and *18S* did not differ between the groups when normalized by each other (Fig. 3).

**Table 1:** The best combination of housekeeping genes for each group of samples yielded by software analysis.

Groups	NormFinder	GeNorm	RefFinder	ΔCt method	Bestkeeper	DataAssist	Best HKG Pair
All	*Ppia/Gapdh*	*Hprt/18S*	*Ppia/18S*	*Ppia/Gapdh*	*18S/Hprt*	*Ppia/Gapdh*	*Ppia/Gapdh* or *Ppia/18S*
Control	*Gapdh/Ppia*	*Gapdh/Ppia*	*Gapdh/Ppia*	*Gapdh/Ppia*	*18S/Hprt*	*Ppia/Gapdh*	*Gapdh/Ppia*
TGF-β	*Ppia/Gapdh*	*18S/Hprt*	*18S/Ppia*	*Ppia/18S*	*18S/Hprt*	*Ppia/Gapdh*	*Ppia/18S*

All, all samples. HKG, housekeeping genes.

### Determination of the suitable number of housekeeping genes

**Figure 4:**
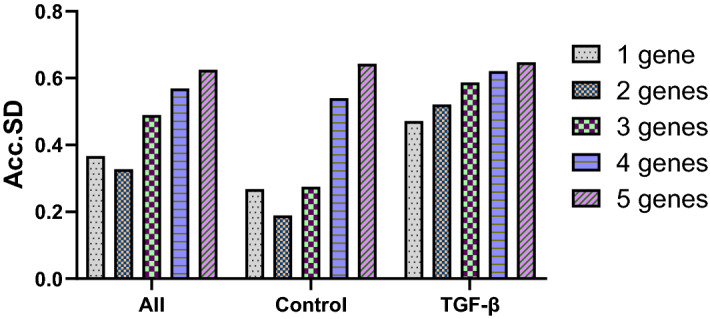
Optimal number of housekeeping genes according to GenEx software analysis. Accumulated standard deviation (Acc.SD) for the five candidate reference genes in all groups to estimate the ideal number of genes for normalization. Lower values of Acc.SD indicate the optimal number of reference genes. All, all samples.

After rating the candidate HKG by their stability values, the optimal number of candidate genes to be used in each dataset must be established. The Acc.SD results showed that one gene (*Ppia* or *18S*) is the optimal number of HKG for normalization of gene expression in TGF-β-treated samples (Fig. 4). Two genes are required for normalization in the All group (*Ppia* + *Gapdh* or *Ppia* + *18S*) and in the control group (*Gapdh* + *Ppia*) (Fig. 4).

### Correlation between the top three candidate housekeeping gene expressions

**Figure 5:**
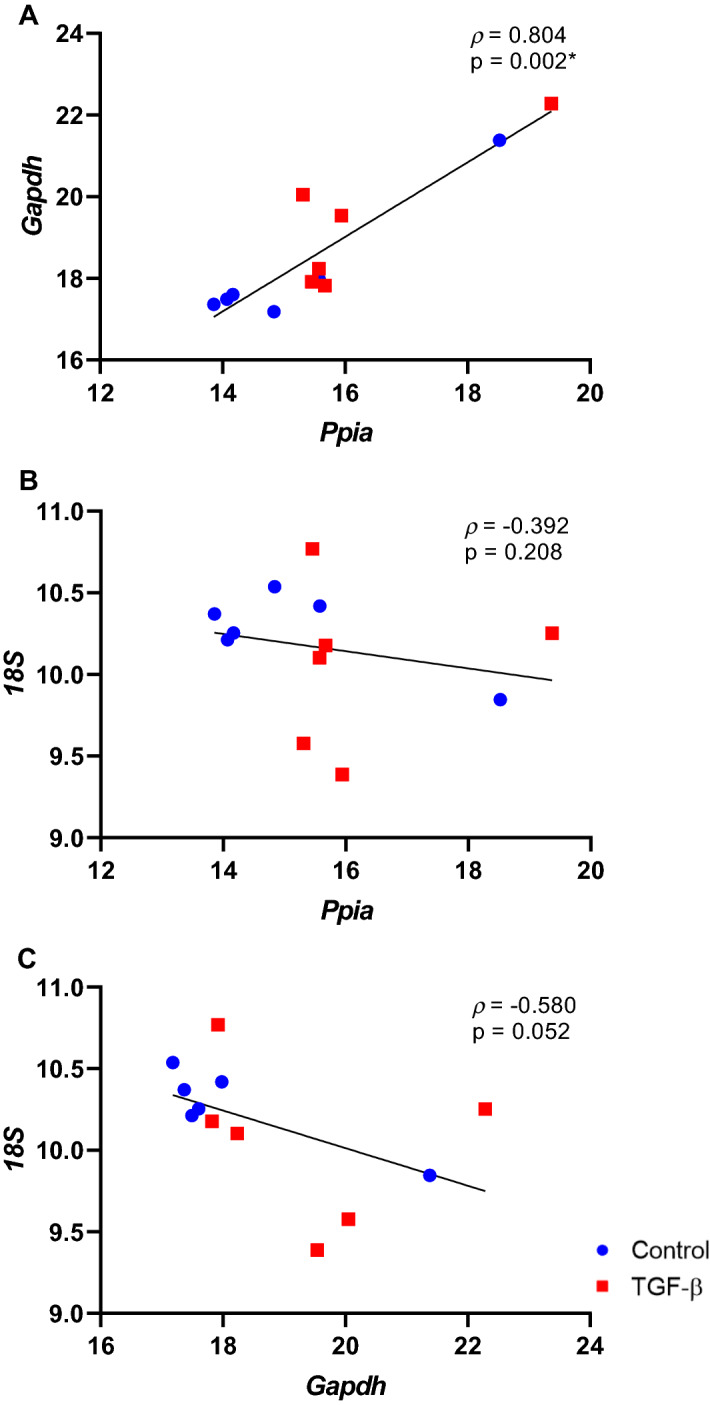
Correlation matrix between the expression of *Ppia* and *Gapdh* (**A**), *Ppia* and *18S* (**B**) and *Gapdh* and *18S* (**C**) candidate housekeeping genes. *ρ*: Spearman's rank correlation coefficient. *p < 0.05.

Correlation analysis were performed using the gene expression data from all samples. The expression levels of the three best candidate HKG showed a strong correlation between *Ppia* and *Gapdh* (*ρ* = 0.804, p = 0.002) (Fig. 5). Also, no statistically significant correlation was found between *Ppia* and *18S* (*ρ* = −0.392, p = 0.208) and *Gapdh* and *18S* (*ρ* = −0.580, p = 0.052) (Fig. 5). These results suggest that besides *Ppia* and *Gapdh* showed a strong correlation, they are correlated in all the samples and can be used together as suitable HKG.

### Validation of the best candidate housekeeping genes for normalizing target genes of *fibronectin, vimentin,* and *α-SMA*

**Figure 6:**
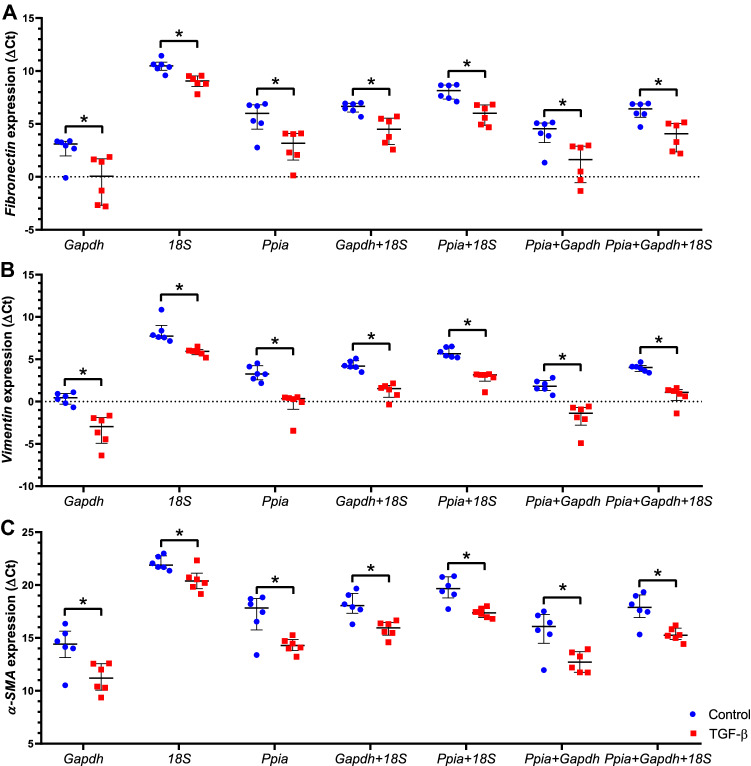
The ΔCt values of *fibronectin* (**A**), *vimentin* (**B**), and *α-SMA* (**C**) target genes normalized by different combinations of the three best candidate housekeeping genes (*Ppia*, *Gapdh,* and *18S*). A negative ΔCt value indicates that the target gene is more abundant than the HKG. The median values are expressed as horizontal lines, and the error bars represent the interquartile range. p < 0.05 by Mann–Whitney *U* test: * vs control group.

To validate the stability of the top three candidate HKG, the relative expression of *fibronectin*, *vimentin,* and *α-SMA* target genes was normalized using different combinations of *Ppia*, *Gapdh* and *18S* (Fig. 6). The expression levels of *fibronectin*, *vimentin,* and *α-SMA* target genes were consistent with upregulation in the TGF-β group relative to controls (Fig. 6). The normalization of target genes by the two less stable genes (*Actb* and *Hprt*) was also evaluated (Supplementary Fig. 1). The use of these HKG, whether alone or in combination, was not able to demonstrate the statistically significant difference that was expected between controls and TGF-β-treated samples of *fibronectin* and *α-SMA* (Supplementary Fig. 1).

## Discussion

The broadly accepted method used to normalize gene expression through RT-PCR technology involves the expression of endogenous HKG. However, the utility of HKG must be validated for specific experimental conditions, since the expression of these endogenous genes can vary depending on experimental conditions^8–10,15^. In vitro systems, including cultured MCs, constitute an useful model to study many pathophysiological states affecting the glomeruli, such as glomerulosclerosis^1^. Therefore, we aimed to determine the most stable reference genes for mRNA quantification in studies performed in vitro*,* mimicking the in vivo glomerular fibrosis using MMCs treated with TGF-β^10,13,15,30^.

Since each algorithm ranked the best candidate HKG, the software packages recommended *Ppia*, *Gapdh* and *18S* as the most stable reference genes between the groups. Peptidylprolyl isomerase A (*Ppia*), a highly abundant protein in the cytoplasm, takes part in various intracellular functions, including a homeostatic role in protein folding and trafficking, intracellular signaling, transcription, inflammation, apoptosis, and regulation of other proteins^31–33^. Glyceraldehyde-3-phosphate dehydrogenase (*Gapdh*) catalyzes the sixth reaction of anaerobic glycolysis, which produces ATP and pyruvate. Other than metabolic functions, this enzyme has been implicated in non-metabolic processes, such as apoptosis induction, DNA repair, cellular proliferation, and transcriptional activation^34–36^. Small subunit 18S ribosomal RNA (*18S*) is the smallest component of eukaryotic cytoplasmic ribosomes and is used as one of the molecular markers^37,38^.

The other two genes considered in this study (*Hprt* and *Actb*) are also commonly used as reference genes. Hypoxanthine phosphoribosyltransferase (*Hprt*) is responsible for purine metabolism, and deficiency of this gene dysregulates cell cycle-controlling functions and cell proliferation mechanisms^39,40^. Actin beta (*Actb*), which is highly abundant in eukaryotic cells, is essential for a variety of cellular functions and is involved in maintaining the cell’s structure, integrity, and motility^41^. Although extensively used as reference genes^14^, *Hprt* and *Actb* ranked as the least stable in this study; however, further studies are needed to better delineate the interactions of these genes with TGF-β.

Since *Ppia*, *Gapdh,* and *18S* were the most suitable candidate reference genes, we normalized them by each other, resulting in no statistically significant differences between groups, which suggests that these genes are good choices for our experimental conditions. After determining the candidate HKG by their stability values, we established the optimal number of reference genes using GenEx software. According to calculated Acc.SD, the optimal number of HKG in this model is the combination of two genes. When used together, *Ppia* and *Gapdh* showed a strong correlation, indicating that all samples were correlated and validating the best pair of HKG.

It is well demonstrated that TGF-β stimulates production of *fibronectin*, *vimentin,* and *α-SMA* in cultured MCs^2,42^; thus, the best HKG combinations herein determined were used to normalize these target genes. Several studies have reported that *Ppia*^14,17,43–48^, *Gapdh*^44,49,50^, and *18S*^45,51–53^ are suitable reference genes and could be used as normalizers of target genes in different models. In the present study, the top three candidate reference genes, whether used alone or in combination, showed the expected increase in the expression of the target genes in the TGF-β-treated group. In contrast, the less stable HKG, employed alone or in combination, did not yield these expected differences, indicating that the in silico analysis selected the better, more stable HKG for this in vitro fibrosis model; they also revealed that an inadequate choice of the endogenous standard HKG could influence the results.

It is important to mention that other non-tested genes can also be used for normalization of the expression of target genes, and additional studies are needed to identify additional candidate genes. Furthermore, this study is specific to MMCs stimulated with TGF-β; thus, the conclusions drawn from our study are not transferable to other models that employ MMCs.

Validating gene expression stability of reference genes is crucial for reliable normalization of RT-PCR data. The work herein presented will serve as a reference for future studies using MMCs stimulated with TGF-β and allow a higher reliability and reproducibility in the identification of gene expression alterations.

Among the reference genes tested in this study, the combination of *Ppia* and *Gapdh* was the best HKG pair and should, therefore, be used as HKG in gene expression analysis in TGF-β-treated MMCs models.

## Methods

### Mesangial cell culture

Mouse mesangial cells (MMCs) were purchased from ATCC (CRL 1927), and the recombinant mouse transforming growth factor-beta (TGF-β) was obtained from R&D Systems (USA). MMCs were grown at 37ºC in plastic flasks in Dulbecco’s Modified Eagle’s medium/Ham’s F12 Medium (DMEM/F12; 3:1 mixture; Invitrogen Corporation, Gaithersburg, MD, USA) containing 10% fetal bovine serum (FBS), penicillin (50 U/mL), and 2.6 g HEPES. The culture flasks were maintained in a 95% air atmosphere and 5% CO_2_ humidified environment. At confluence, cells were exposed to DMEM/F12 medium containing no FBS for 24 h according to the following experimental groups: control, cultured in DMEM/F12 medium and TGF-β group, cultured in DMEM/F12 containing 5 ng/mL of recombinant TGF-β. After 24 h of incubation, cells were rinsed twice with PBS, and 1 mL of the commercial kit (TRIzol, Gibco BRL, Rockland, MD, USA) was added to isolate total RNA and evaluate the mRNA expression.

### Housekeeping genes

The selection of the candidate HKG was based on previous studies that used RT-PCR for gene expressions in kidney diseases models. Then, five genes were selected (*Actb, Hprt, Gapdh, 18S* and *Ppia)* being constitutively expressed in kidney cells with independent cellular functions.

### mRNA expression by RT-PCR

The mRNA expression levels were estimated by quantitative RT-PCR. The total RNA was purified from MMCs using TRIzol kit according to the manufacturer’s instructions. The RNA quantity and purity were determined using the NanoVue spectrophotometer (GE Healthcare Life Sciences, USA). A mass of 2 µg of total RNA was treated with DNase (Promega, Madison, WI, USA) to prevent genomic DNA contamination and DNase inactivation was performed according to manufacturer’s instructions. The RNA pellet was resuspended in RNase-free water and reverse transcribed into cDNA using a High-Capacity cDNA Reverse Transcription Kit (Applied Biosystems). RT-PCR amplification was performed in triplicate using SYBR Green (Applied Biosystems) in the QuantStudio (TM) 7 Flex System (Applied Biosystems), with specific primers for each gene as follows (sense and antisense, respectively): *Fibronectin* (5'’ acactaacgtaaattgcccca 3’ and 5’ gctaacatcactggggtgtggat 3’), *Vimentin* (5’ aggtggatcagctcaccaatgaca 3’ and 5’ tcaaggtcaagacgtgccagagaa 3’), *α-SMA* (5’ tattgtgctggactctggagatgg 3’ and 5’ agtagtcacgaaggaatagccacg 3’), *Actb* (5’ cctcatgccaacacagtgc 3’ and 5’ acatctgctggaaggtggac 3’), *Hprt* (5’ ctcatggactgattatggacaggac 3’ and 5’ gcaggtcagcaaagaacttatagcc 3’), *Gapdh* (5’ ggtggtctcctctgactttaaca 3’ and 5’ accaggaaatgagccttgacaaag 3’), *18S* (5’ gactgtctcgccggtgtc 3’ and 5’ ggagagccggaacgtcga 3’) and *Ppia* (5’ caggtccatctacggagaga 3’ and 5’ catccagccattcagtcttg 3’). The relative gene expression was calculated using the PCR conditions under which the amplification curve was logarithmic.

### Analysis of housekeeping gene expression stability

To define the best housekeeping gene and the best combination, we evaluate the cycle thresholds (Ct) value of RT-PCR in five different software applications: DataAssist (version 3.1; https://www.thermofsher.com/br/en/home/technical-resources/sofware-downloads/dataassist-sofware.html), Bestkeeper (version 1.0; https://www.gene-quantifcation.de/bestkeeper.html), RefFinder and the comparative ΔCt method (https://www.heartcure.com.au/refnder/), GeNorm (https://genorm.cmgg.be/), and NormFinder (version 0.953; https://moma.dk/normfnder-sofware), following the authors’ recommendations. These software packages determine the relative expression stability of the candidate HKG and generate a rank of the best genes^16^. NormFinder is a freely available tool and recommends a standard deviation less than 0.5 (SD < 0.5). GeNorm software calculates the gene stability measure (M value) and recommends that this value falls below 1.5 (M < 1.5). BestKeeper evaluates the SD and coefficient of variation (CV) of the samples, and a SD of less than 1.0 (SD < 1.0) and a CV of less than 3.0 (CV < 3.0) are required. DataAssist shows the Ct values of the candidate genes for all samples and organizes them by score. The comparative ΔCt method was used to calculate the mean SD of the samples. RefFinder software includes all the above software and calculates the geometric mean (Geomean). The optimal number of HKG was evaluated using the GenEx software package, which calculates the accumulated standard deviation (Acc.SD) of sample groups and estimates the ideal number of genes for normalization. The following groups were evaluated: control, TGF-β-treated and All (control + TGF-β samples).

### Statistical analysis

The test of normality (Shapiro–Wilk test) showed that Ct values of HKG were not normally distributed; hence, the median values are expressed as horizontal lines, and the error bars represent interquartile range. All groups were analyzed using the Mann–Whitney *U* test and the Spearman's correlation. Values between 0.30–0.50 were considered as a weak correlation, 0.50–0.70 as moderate, 0.70–0.90 as strong and 0.90–1.00 as very strong correlation.

## Supplementary Information

Supplementary Table 1.

Supplementary Figure 1.

Supplementary Legends.

## Data Availability

All data including supporting datasets are made available as main figures or Supplementary Information Files.
