# Supplementary figures and images for: Determination of reference genes as a quantitative standard for gene expression analysis in mouse mesangial cells stimulated with TGF-β
Source: Sci Rep. 2022 Sep 17;12:15626. doi: 10.1038/s41598-022-19548-z (PMC9482652; doi:10.1038/s41598-022-19548-z)

**A**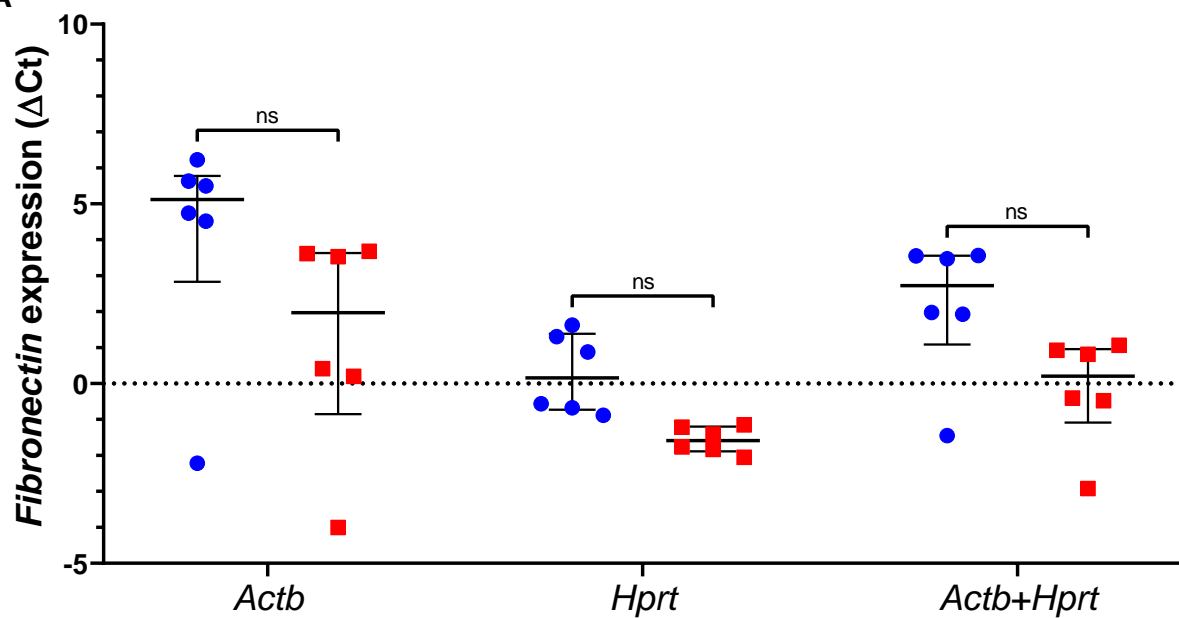**B**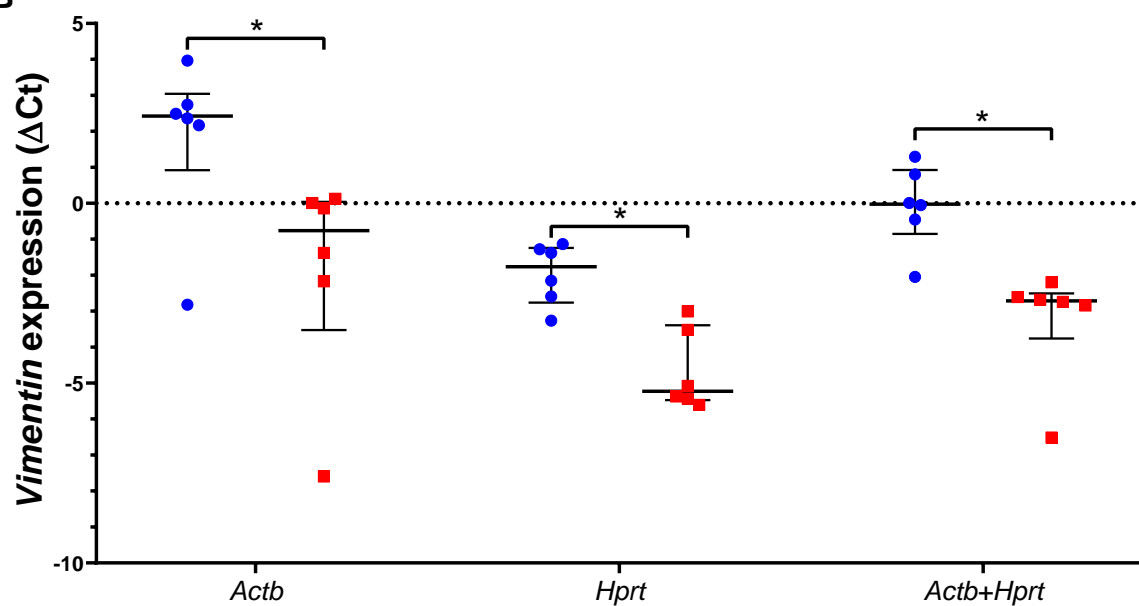**C**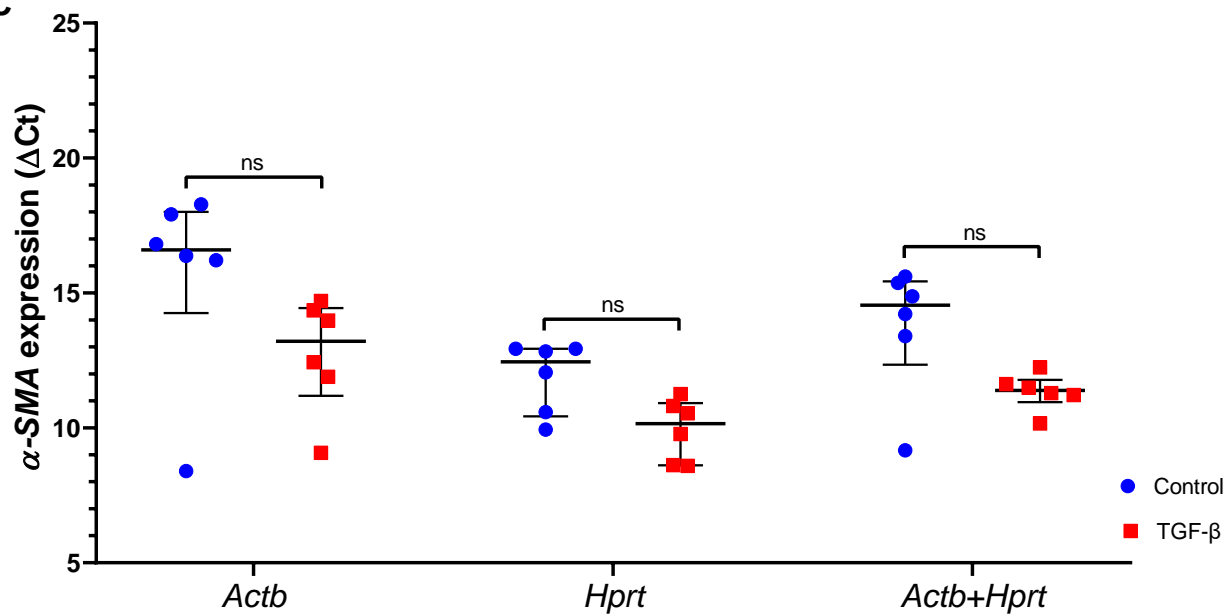

Supplement: Supplementary file 2 — Supplementary Figure 1. [file 41598_2022_19548_MOESM2_ESM.pdf]
