# Supplementary material for: Determination of reference genes as a quantitative standard for gene expression analysis in mouse mesangial cells stimulated with TGF-β
Source: Sci Rep. 2022 Sep 17;12:15626. doi: 10.1038/s41598-022-19548-z (PMC9482652; doi:10.1038/s41598-022-19548-z)
Supplement: Supplementary file 3 — Supplementary Legends. [file 41598_2022_19548_MOESM3_ESM.docx]

**Supplementary Figure 1.** The ΔCt values of *fibronectin* (**A**), *vimentin* (**B**) and *α-SMA* (**C**) target genes normalized different combinations of the candidate housekeeping genes (*Actb* and *Hprt*). A negative ΔCt value indicates that the target gene is more abundant than the HKG. The median values are expressed as horizontal lines, and the error bars represent the interquartile range. p<0.05 by Mann-Whitney *U* test: * vs control group. ns, non-significant.
